# Hybridization vs decoupling: influence of an h-BN interlayer on the physical properties of a lander-type molecule on Ni(111)

**DOI:** 10.3762/bjnano.11.101

**Published:** 2020-08-04

**Authors:** Maximilian Schaal, Takumi Aihara, Marco Gruenewald, Felix Otto, Jari Domke, Roman Forker, Hiroyuki Yoshida, Torsten Fritz

**Affiliations:** 1 Institute of Solid State Physics, Friedrich Schiller University Jena, Helmholtzweg 5, 07743 Jena, Germany; 2 Graduate School of Engineering, Chiba University, 1-33, Yayoi-cho, Inage-ku, Chiba 263-8522, Japan; 3 Molecular Chirality Research Center, Chiba University, 1-33, Yayoi-cho, Inage-ku, Chiba 263-8522, Japan

**Keywords:** buried interface, decoupling, hexagonal boron nitride, hybridization, tetraphenyldibenzoperiflanthene (DBP), two-dimensional materials

## Abstract

2D materials such as hexagonal boron nitride (h-BN) are widely used to decouple organic molecules from metal substrates. Nevertheless, there are also indications in the literature for a significant hybridization, which results in a perturbation of the intrinsic molecular properties. In this work we study the electronic and optical properties as well as the lateral structure of tetraphenyldibenzoperiflanthene (DBP) on Ni(111) with and without an atomically thin h-BN interlayer to investigate its possible decoupling effect. To this end, we use in situ differential reflectance spectroscopy as an established method to distinguish between hybridized and decoupled molecules. By inserting an h-BN interlayer we fabricate a buried interface and show that the DBP molecules are well decoupled from the Ni(111) surface. Furthermore, a highly ordered DBP monolayer is obtained on h-BN/Ni(111) by depositing the molecules at a substrate temperature of 170 °C. The structural results are obtained by quantitative low-energy electron diffraction and low-temperature scanning tunneling microscopy. Finally, the investigation of the valence band structure by ultraviolet photoelectron spectroscopy shows that the low work function of h-BN/Ni(111) further decreases after the DBP deposition. For this reason, the h-BN-passivated Ni(111) surface may serve as potential n-type contact for future molecular electronic devices.

## Introduction

The interfaces between organic molecules and metal contacts play a crucial role in the design of new molecular electronic devices since they affect the charge carrier injection and therefore the device efficiency. An important process to consider is the electronic interaction of organic molecules that are in direct contact with the metal, i.e., the interaction of frontier orbitals with the bands of the metal substrate, which results in changes of the intrinsic optical and electronic properties of the adsorbed molecule. This process is referred to as hybridization, which may be accompanied by the reduction of the HOMO–LUMO gap, the change of the energy-level alignment, and even charge transfer [1–2]. Some applications, however, require to preserve the intrinsic properties of the molecules such as the typically rather narrow optical absorption and/or emission bands. To achieve this, one needs to electronically decouple the molecules from the substrate, which can be achieved through different ways such as the usage of wide-band-gap insulator thin films (e.g., oxides, alkali halides) [3–4], a molecular spacer layer [5–6], or sp^2^-hybridized two-dimensional interlayers (e.g., graphene and hexagonal boron nitride (h-BN)) [7–8]. The advantageous properties of an h-BN monolayer on metal single crystals are the high crystal quality, chemical inertness and the wide band gap of approx. 6 eV, which apparently renders h-BN a promising candidate for the decoupling of highly ordered molecular films [9–10].

However, indications for a significant hybridization of organic molecules on h-BN/Cu(111) were found recently [11–12]. This raises the question under which specific conditions an h-BN monolayer is sufficient to efficiently decouple organic molecules. Until now only a few publications exist which are concerned with this issue [13–16].

In this work we report on the decoupling process by a direct comparison of tetraphenyldibenzoperiflanthene (DBP) adsorbed on Ni(111) with and without an h-BN interlayer. The latter is known to form a commensurate overlayer in which nitrogen and boron atoms occupy top and fcc hollow adsorption sites, respectively [17]. For this reason, h-BN on Ni(111) exhibits an atomically flat morphology [18–19]. DBP is a promising molecule in the field of organic electronics, for example, as an electron donor [20–23] or acceptor [24] in organic photovoltaic applications, and as a dopant in organic light emitting diodes [25].

For our comprehensive study we utilized differential reflectance spectroscopy (DRS), low-energy electron diffraction (LEED), low-temperature scanning tunneling microscopy (LT-STM), as well as photoelectron spectroscopy (PES). Our results reveal that DBP on h-BN/Ni(111) is well decoupled from the metal substrate Ni(111). Furthermore, it was possible to obtain large domains of highly ordered molecules by depositing at an elevated substrate temperature of 170 °C.

## Results and Discussion

### Optical spectroscopy

**Figure 1:**
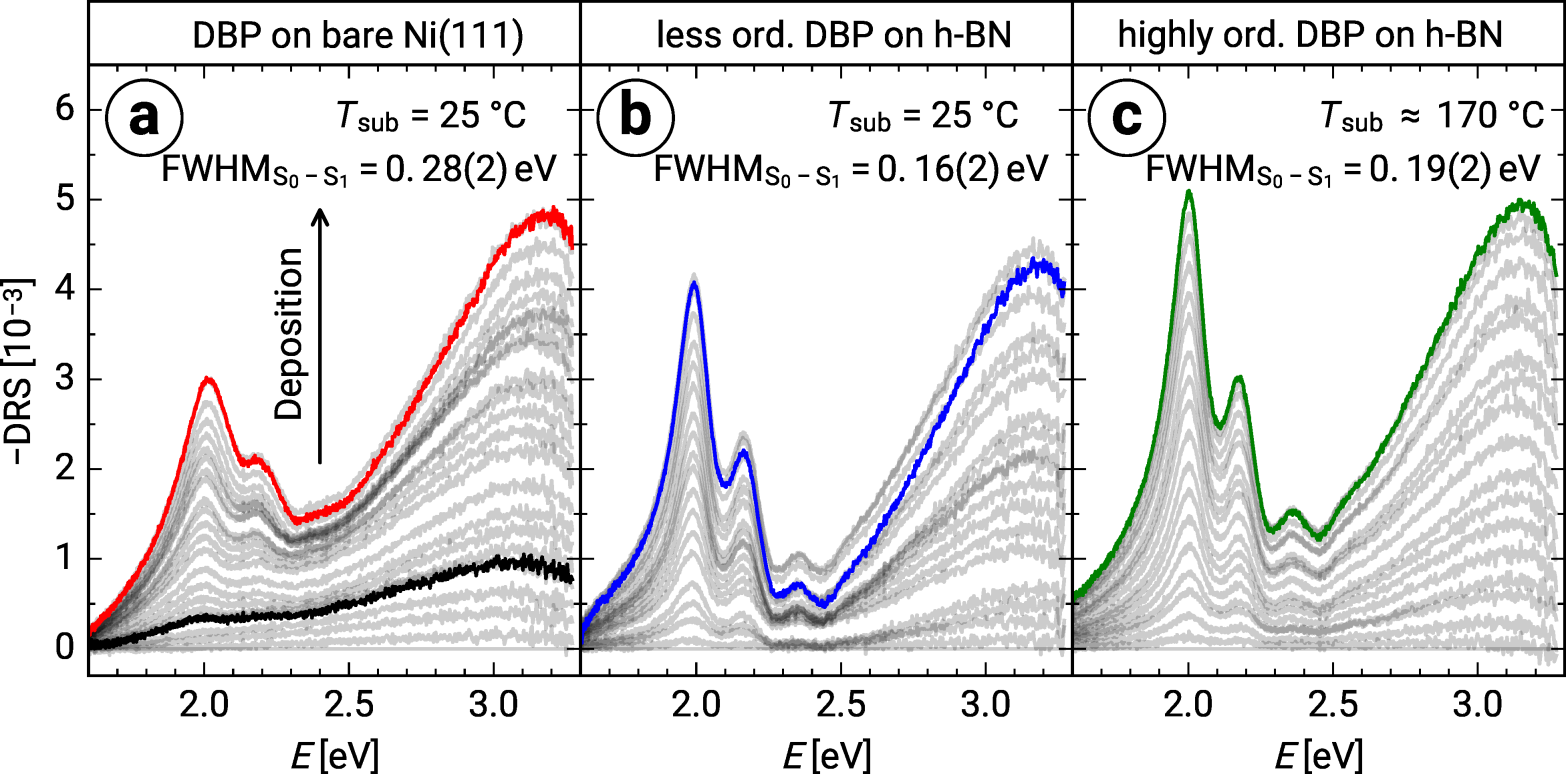
Film-thickness dependent evolution of the DR spectra of (a) DBP on bare Ni(111) as well as of DBP on h-BN/Ni(111) deposited at (b) room temperature and at (c) approx. 170 °C. Colored spectra correspond to a film thickness of 1 MLE. The black spectrum (about 0.25 MLE) is representative of the strong electronic interaction between first-layer molecules and Ni(111). The full widths at half-maximum of the S_0_→S_1_ transitions were determined by a fit of a Voigt function in combination with a linear background.

Figure 1 shows the comparison of the differential reflectance (DR) spectra (definition see Experimental section) of DBP on bare Ni(111) as well as of DBP on h-BN/Ni(111) grown at substrate temperatures *T*_sub_ of 25 °C and 170 °C, respectively. For DBP on Ni(111) deposited at a substrate temperature of 25 °C we observe rather broad and featureless DR spectra at the beginning of the deposition. Such broad spectra are indicative of a strong electronic interaction of first-layer molecules with the Ni(111) substrate. A similar broadening was observed for DBP on Ag(111), where a mixing of the molecular frontier orbitals with the metal bands of the substrate was concluded [26]. After about 0.25 monolayer equivalents (MLE, definition see section “Sample preparation” below), which is marked by a black line in Figure 1, distinct molecular features emerge at about 2.0 and 2.2 eV. We assign these to the S_0_→S_1_ transition and the corresponding vibronic progression of DBP [27]. This indicates an electronic decoupling of the molecules from Ni(111) due to the beginning adsorption already in the second (or higher) layer(s). Further, the somewhat larger peak widths, as compared to the spectra of DBP on mica [25], might be an inhomogeneous broadening effect caused by a higher degree of rotational disorder in the DBP film on Ni(111), compared to DBP on mica [26].

In contrast, the DR spectra of both DBP layers on h-BN/Ni(111) grown at different substrate temperatures show directly the formation of a molecular fingerprint, which is considerably narrower than that of DBP on bare Ni(111) (see full widths at half-maximum (FWHM) of the S_0_→S_1_ transitions in Figure 1) and in very good agreement with the DR spectra of DBP on inert mica [26]. Consequently, h-BN efficiently decouples the molecules deposited on top from the underlying Ni(111) resulting in a monomer-like behavior.

**Figure 2:**
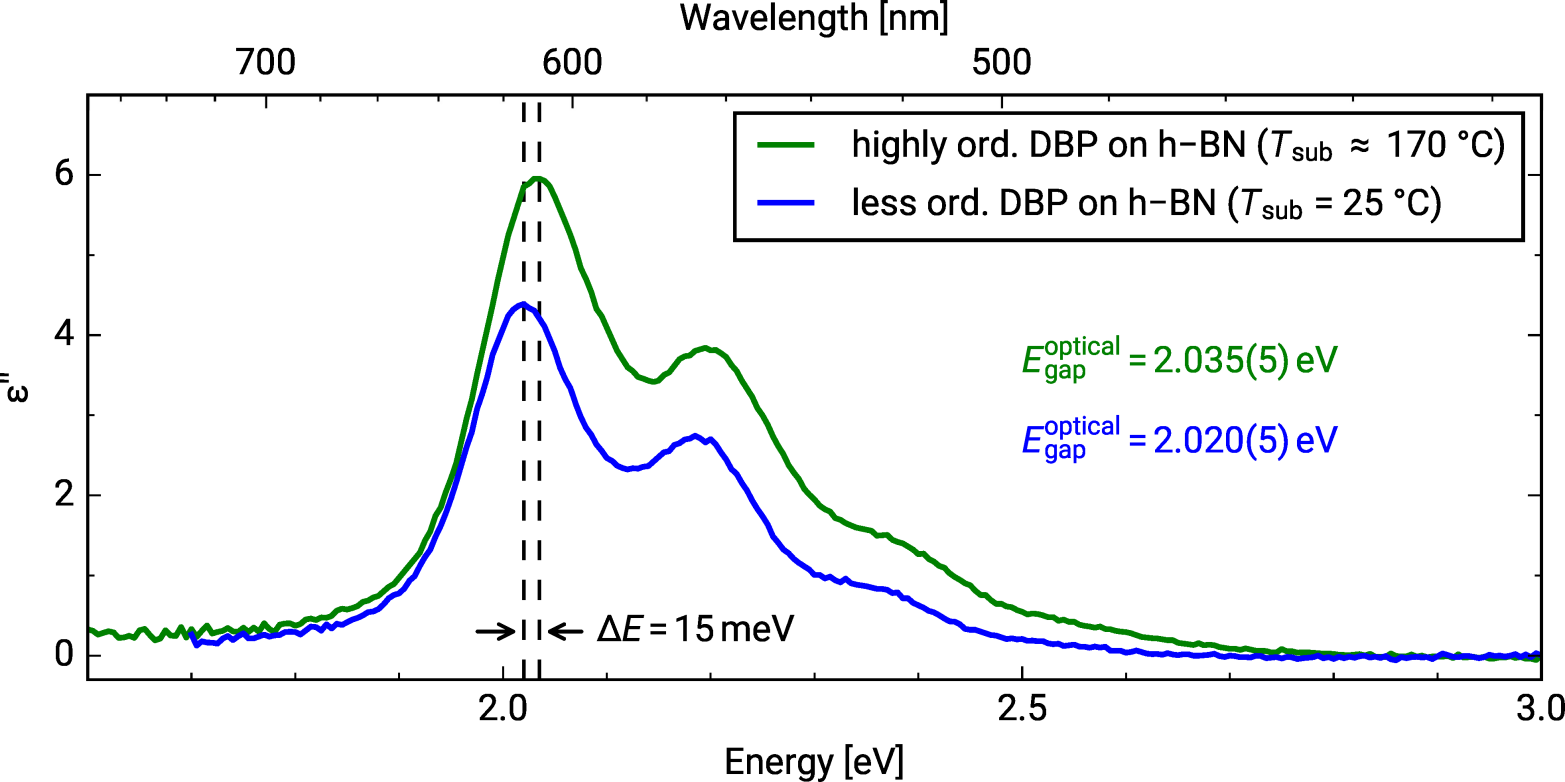
Imaginary part of the dielectric function obtained from the differential reflectance spectra of 1 MLE DBP on h-BN/Ni(111) (blue: substrate temperature = 25 °C, green: substrate temperature approx. 170 °C). Black dashed lines mark the spectral position of the S_0_→S_1_ transition.

Furthermore, we extracted the optical constants from the DRS measurements of both DBP layers on h-BN/Ni(111). The numerical algorithm is described in [28]. In the following, we will focus on the imaginary part of the dielectric function (ε'') only, which is depicted in Figure 2, as this physical quantity is indicative of the optical absorption. The comparison between DBP deposited at substrate temperatures of 25 °C and 170 °C shows larger ε'' values and a slight shift towards higher energies by 15 meV for the latter. Both could be explained by a slightly different packing motif with also different packing densities. The optical gap of DBP adsorbed on h-BN/Ni(111) was determined by the maximum of the absorption peak of the S_0_→S_1_ transition (marked as black dashed lines in Figure 2). Therefore, we obtained values of 2.020(5) and 2.035(5) eV for DBP deposited at substrate temperatures of 25 °C and 170 °C, respectively. The determination of the optical constants of DBP on bare Ni(111) was not possible because of the superposition of the spectra of hybridized molecules and aggregates.

For molecules on top of the first layer the absorption features start to shift to higher energies followed by the formation of a new optical species at lower energies (LE species) as illustrated in Supporting Information File 1, Figure S1. While the shift can be explained by a different dielectric environment of second-layer DBP molecules compared with those in the first layer, the new optical species can be clearly assigned to the fingerprint of DBP aggregates. The similarity of the spectral fingerprints of the monomers in the first and aggregates in higher layers is notable, but can be rationalized by the expected weak excitonic coupling due to the lander-type geometry of DBP. Beside excitonic coupling, also conformational changes may play a role as found for the chemically similar rubrene on highly oriented pyrolytic graphite (HOPG) [29]. Our interpretation of the optical spectra is further supported by LT-STM measurements (see Supporting Information File 1, Figure S2) which show a completely filled monolayer as well as molecular clusters on top of the first layer.

### Lateral structure

In this section we discuss the impact of the h-BN interlayer on the lateral structure of DBP. The LEED measurement of DBP on bare Ni(111) (not shown) exhibits no molecular diffraction pattern, merely a diffuse background is formed upon deposition of DBP. We suggest that the strong interaction of DBP with Ni(111), presumably via the localized d bands, causes a drastic decrease of the mobility of the molecules hindering the highly ordered assembly of the molecules due to a hit-and-stick adsorption. Such a behavior was also observed for one monolayer of pentacene on Ni(111) [30].

In contrast, the LEED measurement of DBP on h-BN/Ni(111) deposited at a substrate temperature of 25 °C shows a ring-like diffraction pattern (see Supporting Information File 1, Figure S3) which can be explained by randomly oriented molecular domains or by a lattice gas or liquid-like phase [31]. A change of the LEED pattern due to a post-growth annealing process in a temperature range from 100 °C to 300 °C was not visible. In fact, at a temperature of 300 °C the desorption of DBP molecules was observed by a decrease of the C 1s intensity measured by XPS (not shown). Therefore, we conclude that a post-growth annealing process does not lead to an increase of the lateral order.

**Figure 3:**
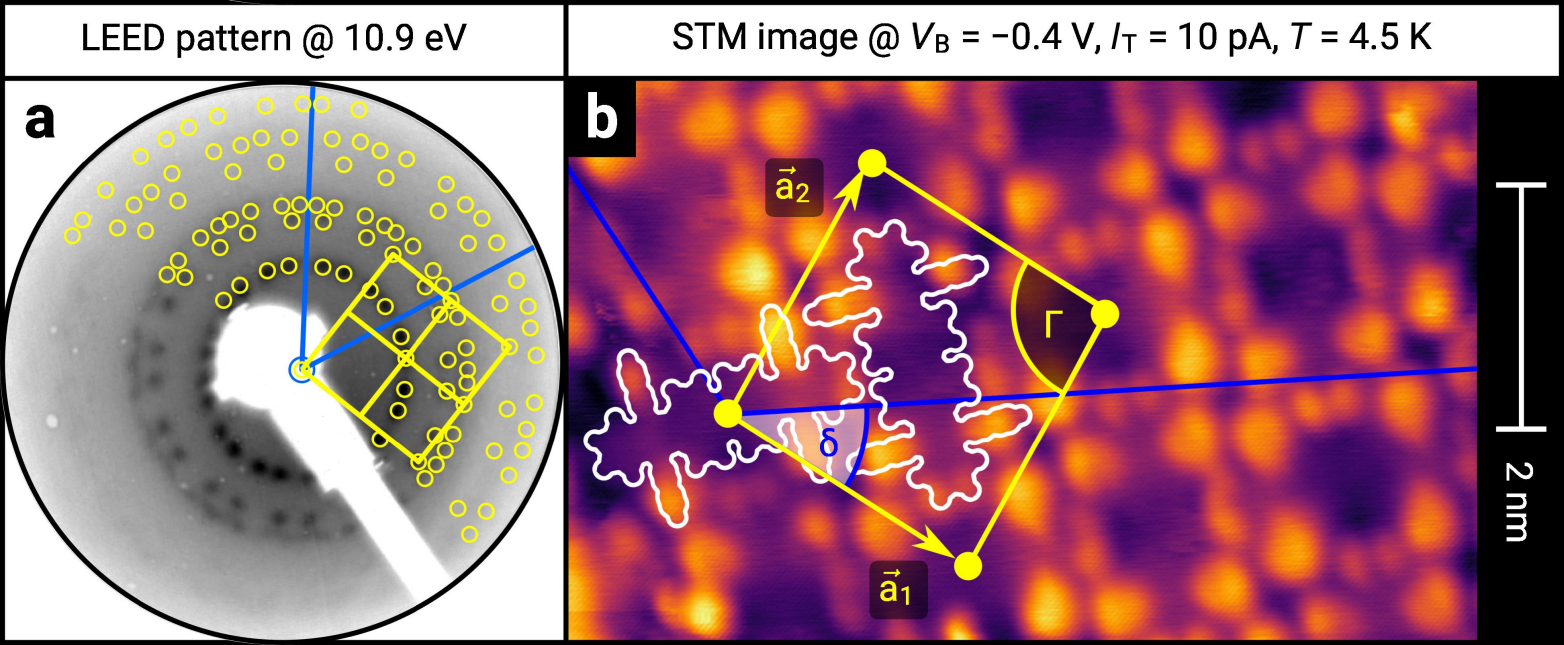
(a) LEED image (logarithmic intensity scale, contrast inverted) of the highly ordered DBP layer on h-BN/Ni(111) grown at a substrate temperature of 170 °C. Half of the LEED image is superimposed by the LEED simulation. Yellow points and lines correspond to the reciprocal lattice of the DBP structure including symmetry equivalents (rotational and mirror domains). Blue lines indicate two primitive reciprocal lattice directions of the substrate. (b) LT-STM image of the same sample, superimposed by the real-space structure of the molecular lattice (marked in yellow) as well as the contours of the two molecules in the unit cell. Blue lines indicate the direction of the primitive lattice vectors of the substrate. The lattice parameters are summarized in Table 1.

However, a highly ordered film was achieved by depositing at a substrate temperature of 170 °C. The LEED image in Figure 3a shows the corresponding diffraction pattern induced by a highly ordered molecular film. For this reason, we labeled this sample as highly ordered DBP layer. The DBP layer that was deposited at a substrate temperature of 25 °C, on the other hand, is labeled as less ordered DBP layer. An increase of the crystal quality of the DBP thin film grown at a substrate temperature higher than 90 °C was also reported by Zhou et al. [32]. The distinct LEED pattern of the highly ordered DBP layer makes it possible to apply a quantitative analysis by means of a LEED simulation that is numerically fitted to the diffraction pattern. The resulting simulation is shown in Figure 3 as yellow circles overlaid with the LEED image. The corresponding epitaxy matrix as well as the lattice parameters are summarized in Table 1.

**Table 1:** Comparison of the lattice parameters obtained by our LEED analysis with reference data of DBP on Au(111) (1 MLE) [33] and on Ag(111) (1.3 MLE) [34]. The angle Γ is defined between the lattice vectors of the adsorbate 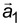 and 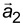. The angle between the adsorbate lattice vector 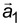 and the direction of the substrate lattice vector 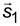 is labeled with δ. Before each epitaxy matrix a prefactor along with its margin of error indicates the absolute scaling uncertainty of the analyzed LEED image. Please note that the epitaxy matrix of DBP on Au(111) and on Ag(111) deviates from the matrix of DBP on h-BN/Ni(111) because of different lattice constants of the substrate as well as slightly different lattice parameters of the adsorbate. We used the acronyms POL and LOL (for point-on-line and line-on-line epitaxy) to characterize the type of epitaxy. The uncertainty of the numerical fitting procedure is given in parentheses behind each value and refers to the last significant digits.

Substrates	| 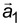 | [Å]	| 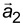 | [Å]	Γ [°]	δ [°]	Epitaxy matrix & type	

h-BN/Ni(111) [this work]	22.8(4)	23.1(5)	90.1(5)	36.1(4)	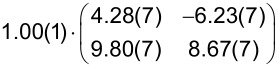	LOL
Au(111) [33]	21.0(3)	23.8(4)	90.2(4)	45.0(3)	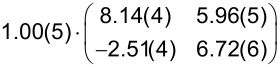	LOL
Ag(111) [34]	20.5(1)	23.2(1)	90.4(1)	−14.1(1)	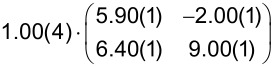	POL

We used the projection method proposed by Forker et al. to identify possible coincidences of the adsorbate and the substrate lattice [35]. We find more than one possible coincidence within the error margin of the epitaxy matrix. However, we can exclude higher-order commensurate (HOC) and point-on-line (POL) coincidences with a substrate order lower than four. By tendency, the higher the substrate order, the lower is the epitaxial energy gain and therefore the probability of the coincidence [35–36]. In the case of highly ordered DBP on h-BN/Ni(111), suitable coincidences with the lowest substrate orders are the on-line coincidences (1, 2), (−1, −2), (−2, 1), and (2, −1).

A comparison with reported lateral structures of DBP on Ag(111) [34] and Au(111) [33] shows very similar adsorbate lattice parameters except for the unit cell rotation with respect to the primitive substrate vector 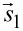 (see Table 1). For this reason, we find that the molecules adopt a similar herringbone arrangement (rectangular unit cell with a basis of two molecules with different azimuthal orientation) on h-BN/Ni(111). This structural model was verified by the LT-STM measurement shown in Figure 3b. We superimposed the STM image by the contours of the two molecules in the unit cell as well as the adsorbate lattice as determined by LEED. A DBP molecule is characterized by four bright protrusions, which correspond to the phenyl substituents oriented nearly perpendicular to the aromatic backbone and two smaller double lobes which correspond to the bisbenz[5,6]indeno end groups [26].

The large-area LT-STM measurement shown in Supporting Information File 1, Figure S2 reveals highly ordered molecular domains with defects at the domain boundaries as well as clusters of molecules on top of the first DBP layer. The fast Fourier transform (FFT) of that STM image resembles the LEED simulation of the molecular lattice (considering eight symmetry equivalent domains only), which supports our structural model.

### Valence band structure and work function change

**Figure 4:**
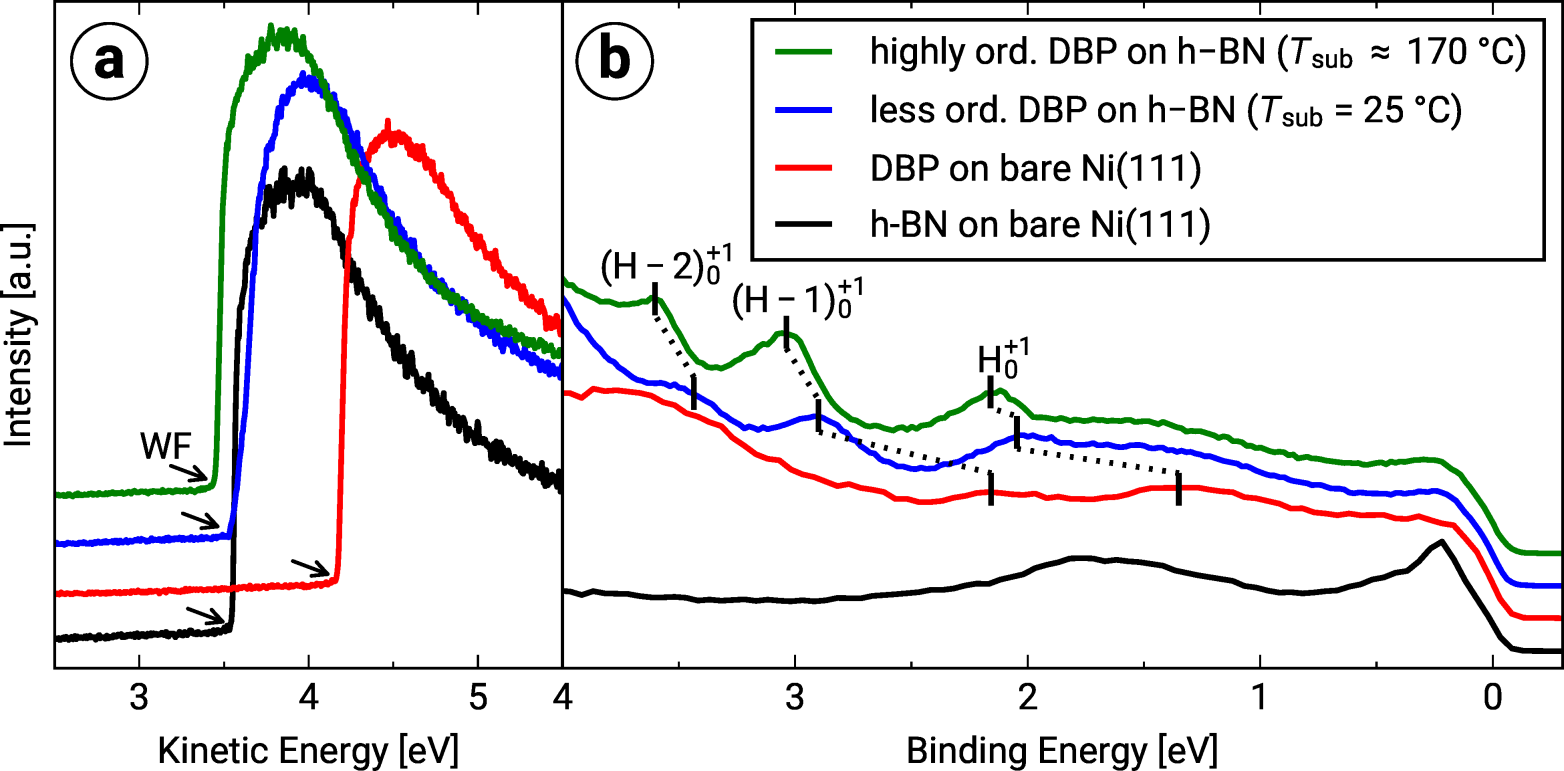
(a) Secondary electron cut-off (SECO) of DBP on bare Ni(111) (approx. 1.0 MLE), on h-BN/Ni(111) (less ordered (approx. 1.0 MLE) and highly ordered (approx. 1.6 MLE)), and of bare h-BN/Ni(111). The kinetic energy onset of the SECO, which corresponds to the work function is marked by black arrows. (b) Corresponding ultraviolet photoelectron spectra at a polar angle of 70°. The positions of the HOMO (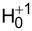), HOMO-1 (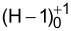) and HOMO-2 (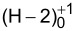) are marked by vertical black lines.

The ultraviolet photoelectron spectroscopy (UPS) measurements of the DBP films on Ni(111) and h-BN/Ni(111) are depicted in Figure 4. We use the notation proposed by Kirchhuebel et al. to assign spectroscopic features to the underlying molecular orbitals, taking into consideration the probing process [37]. Within this notation, each feature is ascribed to the involved molecular orbital with the probing process being characterized by the initial state as subscript and the final state as a superscript. For example, a single-photon photoionization (UPS measurement) describes a transition from the neutral ground state (0) to a positively charged state (+1), namely a cation state. In the case of the HOMO, HOMO-1 and HOMO-2, the notations 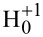, 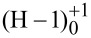, and 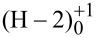 are used, respectively.

The comparison of the different UP spectra shows very broad features for DBP on the bare Ni(111) surface, which in turn renders an identification of the underlying orbitals very difficult. The UPS features of DBP on h-BN/Ni(111), on the other hand, are much sharper and shifted to higher binding energies. The reduction of the line width can be explained by an increase of the structural order, which was already discussed in the last section, as well as by a decrease in hybridization. Probable reasons for the shift of the molecular orbitals to higher binding energies are the work function change as well as the less efficient photo hole screening compared to DBP on bare Ni(111). The adsorption of DBP molecules on the bare Ni(111) surface results in a reduction of the work function from 5.27(2) to 4.17(2) eV. The h-BN layer on Ni(111) causes an even more drastic decrease of the substrate work function to 3.55(2) eV. The subsequent adsorption of DBP on the h-BN interlayer slightly reduces the work function to 3.52(2)/3.45(2) eV (less ordered/highly ordered). The drastic work function reduction for DBP on bare Ni(111) as well as h-BN on Ni(111) results from the strong adsorbate–substrate interaction (hybridization). In contrast, the push-back effect is presumably responsible for the small work function change caused by the adsorption of DBP on h-BN/Ni(111). Furthermore, we determined the adiabatic and vertical ionization energy as distance of the onset and the peak maximum of the HOMO-derived feature to the vacuum level, respectively [38]. Therefore, we used Gaussian fits of the UP spectra in Figure 4. Table 2 summarizes the determined work functions and ionization energies.

**Table 2:** Overview of the work function (WF), adiabatic ionization energy (IE_a_), and vertical ionization energy (IE_v_) for DBP on bare Ni(111) (approx. 1.0 MLE) and DBP on h-BN/Ni(111) (less ordered (approx. 1.0 MLE) and highly ordered (approx. 1.6 MLE)). The uncertainty of the numerical fit is given in parentheses behind each value and refers to the last significant digits. The determination of the adiabatic ionization energy for DBP on bare Ni(111) was not possible because of the overlap of the very broad HOMO-derived feature with the Ni 3d bands.

	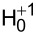 [eV]	WF [eV]	IE_a_ [eV]	IE_v_ [eV]

DBP on Ni(111)	1.35(5)	4.17(2)	–	5.52(7)
less ordered DBP on h-BN/Ni(111)	2.05(1)	3.52(2)	5.26(4)	5.57(3)
highly ordered DBP on h-BN/Ni(111)	2.16(1)	3.45(2)	5.36(4)	5.61(3)

In contrast to Fermi level pinning, where binding energy shifts are not correlated to the work function change, we suggest that the vacuum level alignment is responsible for the energy level alignment since the work function change is sufficient to explain the binding energy shifts of the molecular orbitals. This is a further indication of the weak molecule–substrate interaction and therefore the efficient electronic decoupling of the DBP molecules by the h-BN layer [39–40].

Furthermore, the low work function of h-BN/Ni(111) has quite interesting consequences for possible applications in molecular electronic devices. Low work function metals such as Al, Ca or Ba are typically used to achieve a low electron injection barrier, which is necessary to build high-performance n-type organic semiconductors [41]. However, these substrates suffer from a high chemical reactivity (prone to oxidation) and a strong hybridization with the organic molecules [41]. In contrast to these traditional low work function materials, h-BN on Ni(111) is chemically inert, and we showed that organic molecules like DBP are decoupled from the metal surface which makes this system a promising n-type contact for molecular electronics.

### Core level spectroscopy

**Figure 5:**
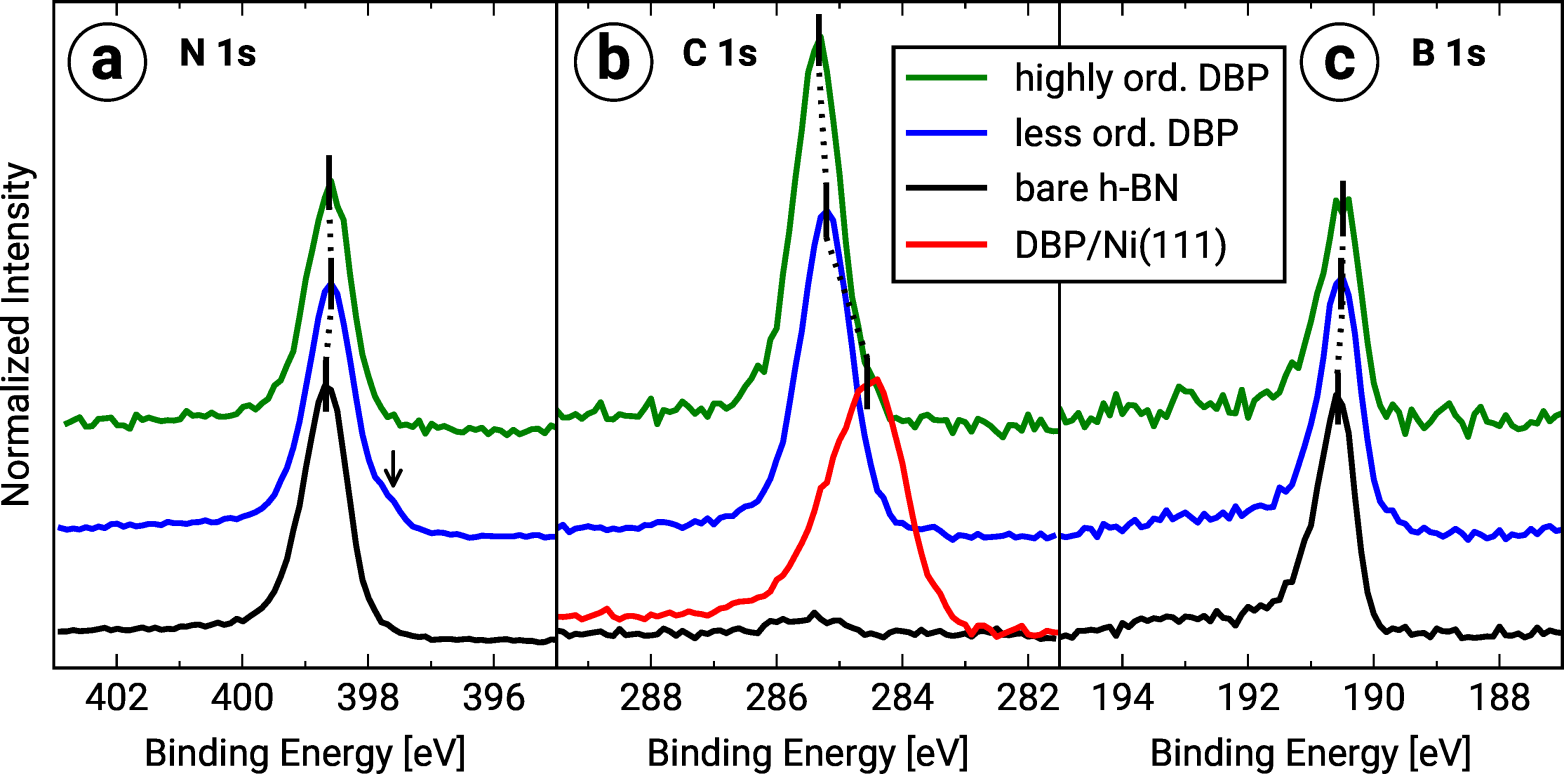
(a) N 1s, (b) C 1s, and (c) B 1s X-ray photoelectron spectra of DBP on bare Ni(111) (approx. 1.0 MLE) as well as of h-BN on Ni(111), and of DBP on h-BN/Ni(111) (less ordered (approx. 1.0 MLE) and highly ordered (approx. 1.6 MLE)). The intensity is normalized to the peak maxima of the N 1s core levels and corrected according to the photoionization cross sections of Yeh and Lindau [44]. For each spectrum the binding energy of the core level is marked by vertical black lines. The black arrow points to an unassigned second component of the N 1s level of the less ordered DBP layer on h-BN/Ni(111).

Finally, we investigated the chemical structure by means of X-ray photoelectron spectroscopy (XPS) at normal emission. In Figure 5 the N 1s, the C 1s and the B 1s spectra for DBP on bare Ni(111) as well as on h-BN/Ni(111) and bare h-BN/Ni(111) are presented. The analysis of the peak positions and intensities was realized by fitting asymmetric pseudo-Voigt functions [42] in the case of the N 1s and the B 1s levels and symmetric pseudo-Voigt functions in the case of the C 1s levels in combination with an active Shirley background [43]. The peak positions of each core level are summarized in Table 3.

**Table 3:** Overview of the binding energies (BE) of the N 1s, C 1s and B 1s core levels for DBP on bare Ni(111) (approx. 1.0 MLE) as well as h-BN on Ni(111) and DBP on h-BN/Ni(111) (less ordered (approx. 1.0 MLE) and highly ordered (approx. 1.6 MLE)). The uncertainty of the numerical fitting procedure is given in parentheses behind each value and refers to the last significant digits.

BE [eV]	N 1s	C 1s	B 1s

DBP on Ni(111)	–	284.56(1)	–
h-BN on Ni(111)	398.67(1)	–	190.58(1)
less ordered DBP on h-BN/Ni(111)	398.59(1)	285.21(1)	190.50(1)
highly ordered DBP on h-BN/Ni(111)	398.62(1)	285.33(1)	190.49(1)

The comparison of the C 1s level of DBP on bare Ni(111) with DBP on h-BN/Ni(111) shows that the peak positions are shifted against each other, and the line width of the latter is significantly reduced. The binding energy shift is consistent with vacuum level alignment (see section “Valence band structure and work function change” above). The origin of the more pronounced broadening as well as the asymmetric line shape [45] of the C 1s level in the case of DBP on bare Ni(111) may stem from a variety of different adsorption configurations (chemical environments) due to disorder and the strong hybridization. We also observed a slight binding-energy shift of the N 1s and B 1s levels after the DBP adsorption, which shows that the DBP molecules also slightly influence the properties of the h-BN interlayer. Furthermore, a small new component at the low binding energy side of the N 1s level (at approx. 397.6 eV) of the less ordered DBP layer is visible. We suggest that the new component may originate from the chemical bonding of the DBP molecules to the nitrogen atoms of the h-BN interlayer, which possibly reduces the molecular mobility. This additional diffusion barrier thus hampers the molecular self-assembly and can be overcome by a higher substrate temperature during the film growth, which agrees with our structural findings above.

## Conclusion

To summarize, we investigated the influence of an h-BN interlayer on the optical, structural and electronic properties of DBP on Ni(111). By inserting the h-BN layer, we fabricated a buried interface, and a monomer-like behavior was observed by means of DRS instead of a strong hybridization, which occurs on the bare metal substrate. Therefore, we conclude that one h-BN layer is sufficient to decouple the DBP molecules from the Ni(111) substrate. This statement is supported by the vacuum level alignment of the frontier orbitals, which was concluded from our UPS data. The investigation of the chemical structure by means of XPS revealed that the DBP adsorption also mildly influences the h-BN interlayer. A notable improvement of the lateral order was achieved by depositing DBP at a substrate temperature of 170 °C. The LEED measurement showed a clear diffraction pattern proving the high ordering of the DBP monolayer. By means of the combination of the quantitative LEED analysis and the LT-STM measurements, we concluded that the DBP molecules adopt a herringbone structure similar to DBP on Ag(111) and Au(111). Furthermore, we observed that the low work function of h-BN/Ni(111) decreases upon DBP deposition down to a value of 3.45(2) eV for the highly ordered DBP layer on h-BN/Ni(111). Therefore, h-BN on Ni(111) can potentially be used as n-type contact in molecular electronic devices with the advantage to minimize the metal–organic hybridization.

## Experimental

### Sample preparation

The Ni(111) single crystal (MaTecK GmbH, Germany, purity 99.99%) was prepared by several cycles of Ar^+^ sputtering at room temperature and annealing at 800 °C. The h-BN layer was grown by thermal dehydrogenation of borazine molecules at a substrate temperature of 800 °C similar to [19]. We purchased borazine from Katchem Ltd. (Czech Republic) with a specified purity of *>*98%. The quality of the h-BN layer was checked by XPS and LEED. DBP raw material was purchased from Luminescence Technology Corp (Lumtec, Taiwan) with a specified purity of *>*99%. To remove remaining impurities we applied two cycles of temperature gradient sublimation according to [46]. The growth of the DBP films was achieved by deposition from an effusion cell at approx. 330 °C in ultrahigh vacuum. The layer thicknesses of the DBP films were determined based on DRS [28]. We use the unit monolayer equivalent (MLE) as defined and calibrated in detail in the Supporting Information File 1, Figure S1. Initially, the DRS signal accumulates rather uniformly for an increasing amount of deposited molecules on the substrate surface. Beginning at a certain threshold the DRS signal exhibits a noticeable blueshift (Supporting Information File 1, Figure S1), and we use this sudden change for our definition of 1 MLE. This spectral shift as well as the subsequent emergence of a new component in the spectra is attributed to DBP adsorbing in the second layer. We emphasize that this definition of 1 MLE does not necessarily imply that the substrate surface is entirely covered with densely packed molecular domains. Yet, we also refer to our large-area STM images (Supporting Information File 1, Figure S2), which confirm a close-packed DBP wetting layer as well as DBP clusters on top for a nominal film thickness of about 1.6 MLE. Hence, there is little discrepancy between 1 MLE, defined via DRS, and a fully covered substrate surface.

### Experimental methods

All experiments were performed in ultrahigh vacuum with a base pressure in the range of 10^−10^ mbar. The adsorption of the DBP molecules was monitored by in situ DRS utilizing a 100 W halogen tungsten lamp, a blazed-grating monochromator (Acton Research SpectraPro SP2156), and a thermoelectrically cooled charge-coupled device (CCD) (Princeton Instruments PIXIS 100BR eXcelon/UV) [28,47–48]. The experimental setup is described in detail in [28]. The DRS signal is defined as:

[1]
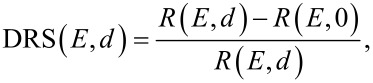


where *R*(*E*, 0) and *R*(*E*, *d*) are the reflectance spectra of the bare substrate and the DBP covered substrate, respectively, and *d* being the film thickness. Each reflectance spectrum was accumulated over 30 s to increase the signal-to-noise ratio. Furthermore, we used the difference of consecutive reflectance spectra to calculate the ΔDRS intensity using the following formula:

[2]
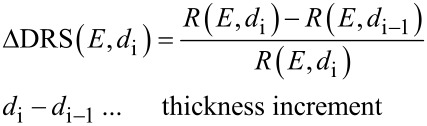


The lateral structure of the DBP films was investigated in reciprocal space using an Omicron MCP-LEED (MCP2-SPECTALEED) and in real space by LT-STM using a JT-STM/AFM (SPECS Surface Nano Analysis GmbH) with a tungsten tip operated at 4.5 K. We used the non-commercial software LEEDCal [49] for the distortion correction of the LEED images and the commercial software LEEDLab [50] for the quantitative LEED analysis. The LT-STM images were only modified by a mean plane subtraction. The electronic properties were investigated by PES with monochromatized Al Kα (SPECS Focus 500, *E*_excitation_ = 1486.71 eV), monochromatized and p-polarized He Iα (SPECS UVLS-600, *E*_excitation_ = 21.22 eV) radiation, and a SPECS PHOIBOS 150 hemispherical electron analyzer equipped with a 3D delay line detector (SPECS DLD4040-150). The energy resolutions of the UPS and XPS measurements were determined to be approx. 10 meV and approx. 0.55 eV, respectively. For the determination of the work function we used a bias voltage of approx. −9 V to shift the secondary electron cut-off.

## Supporting Information

File 1 Additional experimental results.

## References

[1] Duhm S, Gerlach A, Salzmann I, Bröker B, Johnson R L, Schreiber F, Koch N (2008). Org Electron.

[2] Koch N (2008). J Phys: Condens Matter.

[3] Yang X, Krieger I, Lüftner D, Weiß S, Heepenstrick T, Hollerer M, Hurdax P, Koller G, Sokolowski M, Puschnig P (2018). Chem Commun.

[4] Xu C, Que Y, Zhuang Y, Lin Z, Wu X, Wang K, Xiao X (2018). J Phys Chem B.

[5] Forker R, Kasemann D, Dienel T, Wagner C, Franke R, Müllen K, Fritz T (2008). Adv Mater (Weinheim, Ger).

[6] Wang Q, Franco-Cañellas A, Ji P, Bürker C, Wang R-B, Broch K, Thakur P K, Lee T-L, Zhang H, Gerlach A (2018). J Phys Chem C.

[7] Martínez-Galera A J, Nicoara N, Martínez J I, Dappe Y J, Ortega J, Gómez-Rodríguez J M (2014). J Phys Chem C.

[8] Forker R, Dienel T, Krause A, Gruenewald M, Meissner M, Kirchhuebel T, Gröning O, Fritz T (2016). Phys Rev B.

[9] Auwärter W (2019). Surf Sci Rep.

[10] Kratzer M, Matkovic A, Teichert C (2019). J Phys D: Appl Phys.

[11] Wang M, Kim M, Odkhuu D, Park N, Lee J, Jang W-J, Kahng S-J, Ruoff R S, Song Y J, Lee S (2014). ACS Nano.

[12] Jang S K, Youn J, Song Y J, Lee S (2016). Sci Rep.

[13] Brülke C, Heepenstrick T, Krieger I, Wolff B, Yang X, Shamsaddinlou A, Weiß S, Bocquet F C, Tautz F S, Soubatch S (2019). Phys Rev B.

[14] Schwarz M, Duncan D A, Garnica M, Ducke J, Deimel P S, Thakur P K, Lee T-L, Allegretti F, Auwärter W (2018). Nanoscale.

[15] Mehler A, Néel N, Kröger J (2019). J Vac Sci Technol, A.

[16] Matković A, Genser J, Kratzer M, Lüftner D, Chen Z, Siri O, Puschnig P, Becker C, Teichert C (2019). Adv Funct Mater.

[17] Sun X, Pratt A, Li Z Y, Ohtomo M, Sakai S, Yamauchi Y (2014). J Appl Phys.

[18] Auwärter W, Kreutz T J, Greber T, Osterwalder J (1999). Surf Sci.

[19] Preobrajenski A B, Vinogradov A S, Mårtensson N (2004). Phys Rev B.

[20] Hirade M, Nakanotani H, Yahiro M, Adachi C (2011). ACS Appl Mater Interfaces.

[21] Xiao X, Zimmerman J D, Lassiter B E, Bergemann K J, Forrest S R (2013). Appl Phys Lett.

[22] Zheng Y-q, Potscavage W J, Komino T, Hirade M, Adachi J, Adachi C (2013). Appl Phys Lett.

[23] Chen C-W, Huang Z-Y, Lin Y-M, Huang W-C, Chen Y-H, Strzalka J, Chang A Y, Schaller R D, Lee C-K, Pao C-W (2014). Phys Chem Chem Phys.

[24] Bartynski A N, Grob S, Linderl T, Gruber M, Brütting W, Thompson M E (2016). J Phys Chem C.

[25] Nakanotani H, Higuchi T, Furukawa T, Masui K, Morimoto K, Numata M, Tanaka H, Sagara Y, Yasuda T, Adachi C (2014). Nat Commun.

[26] Otto F, Kirchhuebel T, Baby A, Sojka F, Fratesi G, Fritz T, Forker R (2020). J Phys Chem C.

[27] Debad J D, Morris J C, Lynch V, Magnus P, Bard A J (1996). J Am Chem Soc.

[28] Forker R, Gruenewald M, Fritz T (2012). Annu Rep Prog Chem, Sect C: Phys Chem.

[29] Udhardt C, Forker R, Gruenewald M, Watanabe Y, Yamada T, Ueba T, Munakata T, Fritz T (2016). Thin Solid Films.

[30] Dinca L E, De Marchi F, MacLeod J M, Lipton-Duffin J, Gatti R, Ma D, Perepichka D F, Rosei F (2015). Nanoscale.

[31] Bischoff F, Seufert K, Auwärter W, Joshi S, Vijayaraghavan S, Écija D, Diller K, Papageorgiou A C, Fischer S, Allegretti F (2013). ACS Nano.

[32] Zhou Y, Taima T, Shibata Y, Miyadera T, Yamanari T, Yoshida Y (2011). Sol Energy Mater Sol Cells.

[33] Mehler A, Kirchhuebel T, Néel N, Sojka F, Forker R, Fritz T, Kröger J (2017). Langmuir.

[34] Kirchhuebel T, Gruenewald M, Sojka F, Kera S, Bussolotti F, Ueba T, Ueno N, Rouillé G, Forker R, Fritz T (2016). Langmuir.

[35] Forker R, Meissner M, Fritz T (2017). Soft Matter.

[36] Wagner C, Forker R, Fritz T (2012). J Phys Chem Lett.

[37] Kirchhuebel T, Monti O L A, Munakata T, Kera S, Forker R, Fritz T (2019). Phys Chem Chem Phys.

[38] Ivanov M V, Wang D, Zhang D, Rathore R, Reid S A (2018). Phys Chem Chem Phys.

[39] Braun S, Salaneck W R, Fahlman M (2009). Adv Mater (Weinheim, Ger).

[40] Otero R, Vázquez de Parga A L, Gallego J M (2017). Surf Sci Rep.

[41] Quinn J T E, Zhu J, Li X, Wang J, Li Y (2017). J Mater Chem C.

[42] Schmid M, Steinrück H-P, Gottfried J M (2014). Surf Interface Anal.

[43] Herrera-Gomez A, Bravo-Sanchez M, Ceballos-Sanchez O, Vazquez-Lepe M O (2014). Surf Interface Anal.

[44] Yeh J J, Lindau I (1985). At Data Nucl Data Tables.

[45] Klein B P, van der Heijden N J, Kachel S R, Franke M, Krug C K, Greulich K K, Ruppenthal L, Müller P, Rosenow P, Parhizkar S (2019). Phys Rev X.

[46] Levin A A, Leisegang T, Forker R, Koch M, Meyer D C, Fritz T (2010). Cryst Res Technol.

[47] McIntyre J D E, Aspnes D E (1971). Surf Sci.

[48] Sun L D, Hohage M, Zeppenfeld P, Berkebile S, Koller G, Netzer F P, Ramsey M G (2006). Appl Phys Lett.

[49] Sojka F, Meissner M, Zwick C, Forker R, Fritz T (2013). Rev Sci Instrum.

[50] (2018). LEEDLab.

