# Supplementary material for: Hybridization vs decoupling: influence of an h-BN interlayer on the physical properties of a lander-type molecule on Ni(111)
Source: Beilstein J Nanotechnol. 2020 Aug 4;11:1168–77. doi: 10.3762/bjnano.11.101 (PMC7418096; doi:10.3762/bjnano.11.101)
Supplement: File 1 — Additional experimental results. [file Beilstein_J_Nanotechnol-11-1168-s001.pdf]

BEILSTEIN JOURNAL OF NANOTECHNOLOGY

Supporting Information

for

Hybridization vs decoupling: influence of an h-BN interlayer on the physical properties of a lander-type molecule on Ni(111)

Maximilian Schaal, Takumi Aihara, Marco Gruenewald, Felix Otto, Jari Domke, Roman Forker, Hiroyuki Yoshida and Torsten Fritz

Beilstein J. Nanotechnol. **2020,** *11,* 1168–1177. doi:10.3762/bjnano.11.101

Additional experimental results

License and Terms: This is a supporting information file under the terms of the Creative Commons Attribution License (http://creativecommons.org/licenses/by/4.0). Please note that the reuse, redistribution and reproduction in particular requires that the authors and source are credited. The license is subject to the *Beilstein Journal of Nanotechnology* terms and conditions: (https://www.beilstein-journals.org/bjnano)

1. Optical properties of DBP on h-BN/Ni(111) in the transition between mono- and multilayer

In this section we will discuss our results of the layer-thickness-dependent change of the optical properties of DBP on h-BN/Ni(111) deposited at a substrate temperature of approx. 170 °C. This change in combination with the assumption of a constant deposition rate allows for a precise determination of the film thickness. Figure S1 shows the ΔDR spectra of DBP on h-BN/Ni(111) in a thickness range from 0.95 to 1.37 MLE. Initially, we observed a peak shift of the $S_0 \rightarrow S_1$ transition towards higher energies, which is indicated by the thick red lines. The corresponding film thickness of the last unshifted spectrum was defined to 1.00 MLE. In the following we used this definition for the thickness calibration and the assumption of a constant deposition rate to determine the layer thickness of each spectrum. Furthermore, we observed the formation of a new optical species above a layer thickness of 1.21 MLE, which arises at a lower energy compared to the previously discussed species. For this reason, we labeled the new optical species as low-energy (LE) and the initial species as high-energy species (HE). The LE species is marked with the thick blue lines. The description of the origin of the peak shift and the emerging of the LE species are given in the main text.

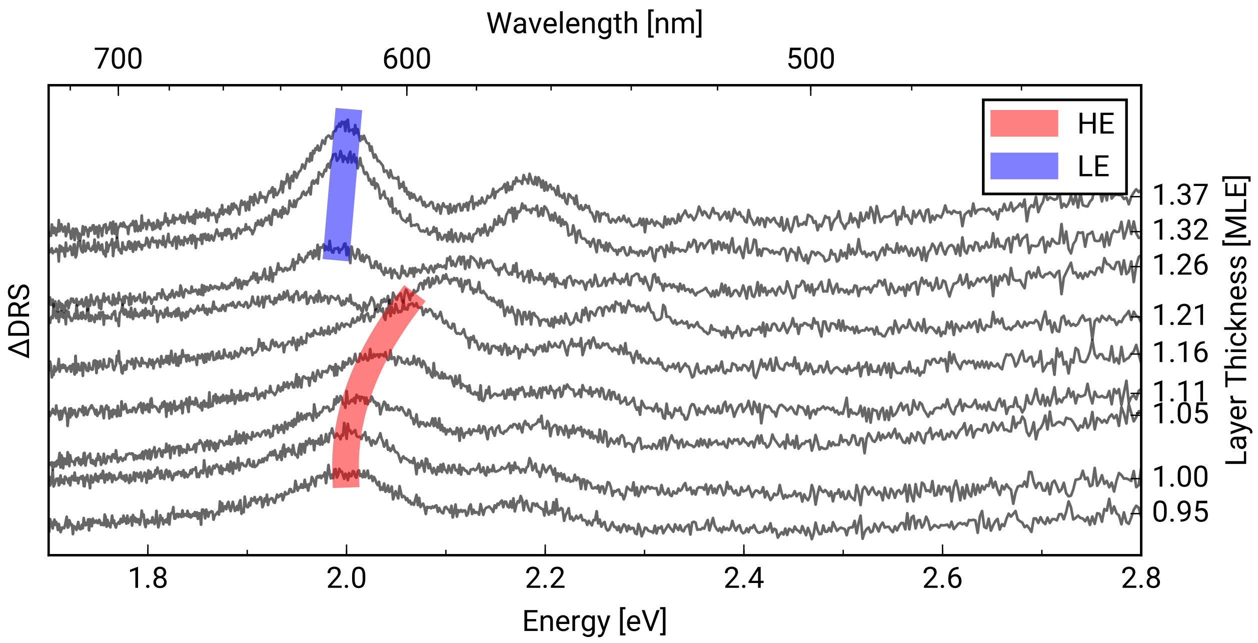

Figure S1: ΔDR spectra of the highly ordered DBP film on h-BN/Ni(111). The spectra are vertically offset for the sake of clarity. Thick red an blue lines are guides to the eye and correspond to the peak positions of a high energy (HE) and a low energy (LE) species. The layer thickness of each spectrum is given on the right axis.

2. Lateral structure of DBP on h-BN/Ni(111) deposited at a substrate temperature of approx. 170 °C

Figure S2 shows a large LT-STM scan (a) and the corresponding FFT (b). The LT-STM image visualizes a highly ordered DBP monolayer consisting of a variety of domains as well as molecular clusters on top of the first layer. Furthermore, defects at the domain boundaries are visible. The FFT shows bright spots that originate from the highly ordered DBP monolayer. We can compare the FFT of the LT-STM image with the LEED simulation (described in the main text), since the real and reciprocal space are linked by a Fourier transform. Therefore, we decreased the number of symmetry equivalent domains in the simulation to eight and scaled it so that it fits the FFT (yellow circles in Figure S2). The comparison shows a quite good agreement.

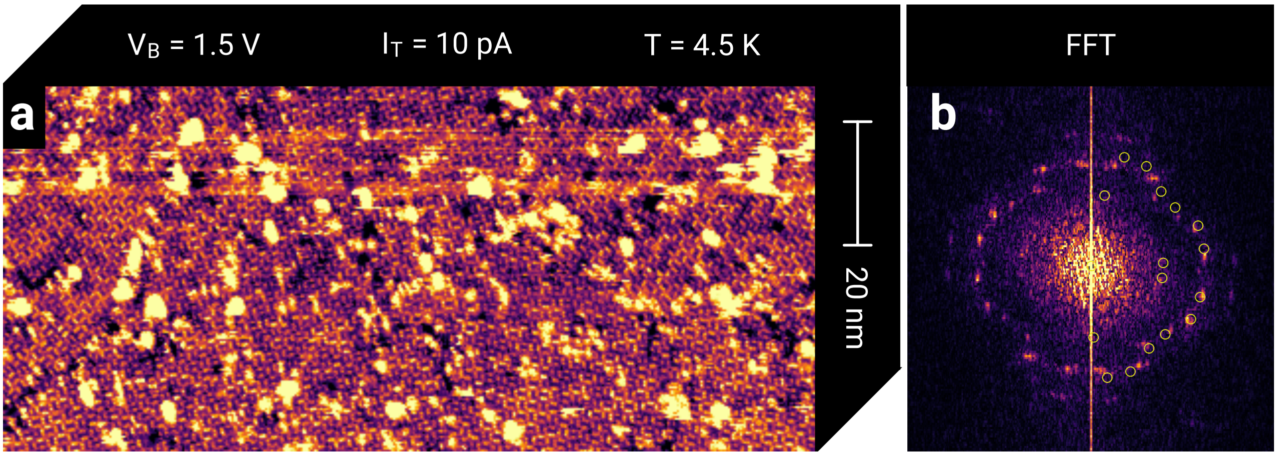

Figure S2: (a) LT-STM scan of the highly ordered DBP film (1.6 MLE) on h-BN/Ni(111) showing large domains of highly ordered molecules as well as defects at the domain boundaries and clusters of molecules in higher layers. (b) FFT of the same STM image is superimposed by the LEED simulation (yellow circles) as discussed in the main text, but considering only eight symmetry equivalent domains.

Lateral structure of DBP on h-BN/Ni(111) deposited at a substrate temperature of 25 and 170 °C

Figure S3 shows the comparison of the LEED pattern of DBP on h-BN/Ni(111) deposited at a substrate temperature of 25 °C (Figure S3a) and 170 °C (Figure S3b). We observed a ring-like diffraction pattern for the DBP layer deposited at room temperature. In contrast, a highly ordered DBP layer was deposited at 170 °C. Furthermore, a quantitative LEED analysis was conducted for the highly ordered DBP layer. The result is shown as yellow circles superimposed on both LEED images. The comparison of the diffraction pattern of the less ordered DBP layer with the LEED simulation shows that the diameter of the ring-like pattern is satisfactorily reproduced by the spots of the LEED simulation. Therefore, we conclude that this originates from a variety of rotational domains similar to the highly ordered structure of DBP on h-BN/Ni(111).

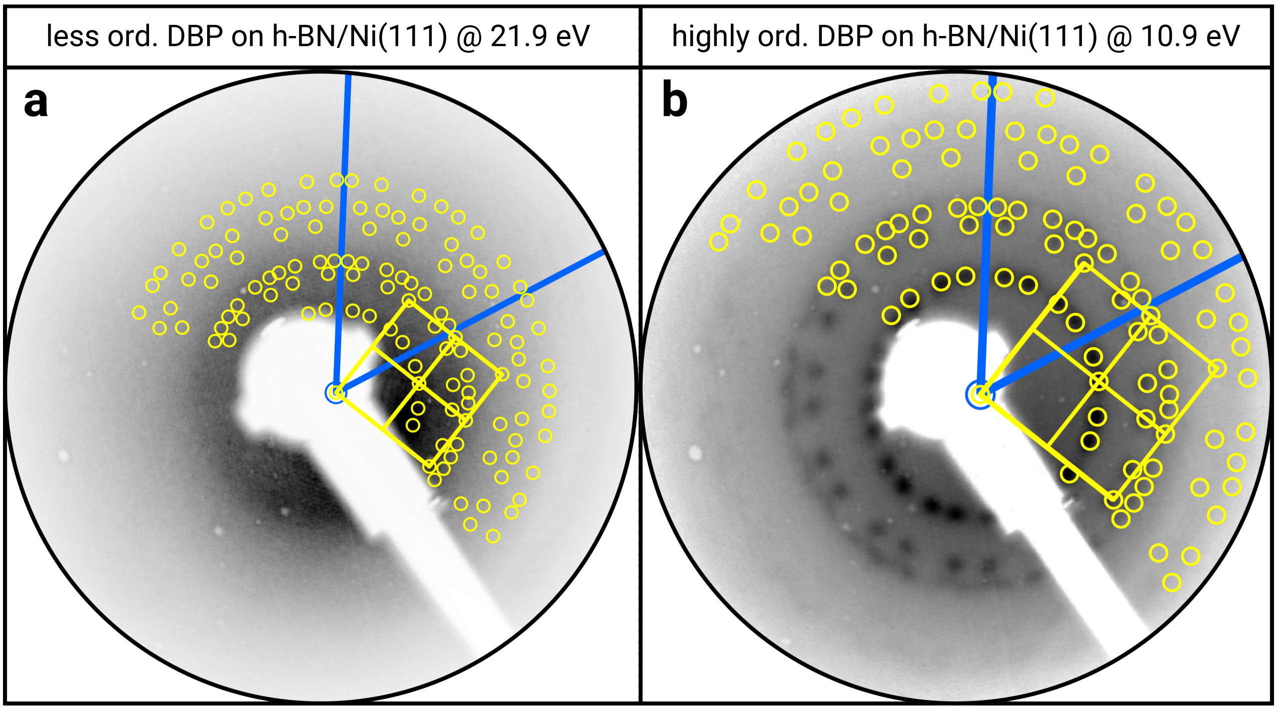

Figure S3: LEED patterns of DBP on h-BN/Ni(111) deposited at a substrate temperature of 25 °C (less ordered, (a)) and 170 °C (highly ordered, (b)). The LEED images are superimposed with the simulation for the highly ordered layer (see main text).
